# Assessment of an Algorithm for Prescription of Oral Anticoagulation for Patients With Atrial Fibrillation in Emergency Departments

**DOI:** 10.1001/jamanetworkopen.2020.0306

**Published:** 2020-03-03

**Authors:** Clare L. Atzema, Jiming Fang, Jafna L. Cox, Alice S. Chong, Karen Tu, Peter C. Austin

**Affiliations:** 1 ICES, Toronto, Ontario, Canada; 2 Sunnybrook Health Sciences Centre, Toronto, Ontario, Canada; 3 Division of Emergency Medicine, Department of Medicine; 4 Institute for Health Policy, Management and Evaluation; 5 Division of Cardiology, Department of Medicine; 6 Department of Community Health and Epidemiology, Dalhousie University, Halifax, Nova Scotia, Canada.; 7 Department of Family and Community Medicine, the University of Toronto, Toronto, Ontario, Canada; 8 University Health Network-Toronto Western Hospital Family Health Team, Toronto, Ontario, Canada

## Abstract

This retrospective study uses a Canadian reporting system to validate an algorithm that identifies oral anticoagulation prescriptions given to patients with atrial fibrillation treated in emergency departments.

## Introduction

Atrial fibrillation is seen commonly in emergency departments (EDs).^1,2^ Initiation of oral anticoagulation to patients treated in the ED has been associated with higher long-term use than when prescribing is left to outpatient care following discharge.^3^

Current data sets do not identify ED prescriptions. The ability to identify these prescriptions in large data sets could facilitate future studies aimed at increasing such prescriptions and could support long-term monitoring of ED oral anticoagulant prescription patterns.

## Methods

This retrospective cohort study sought to validate an algorithm that identifies ED oral anticoagulant prescription provision for patients with atrial fibrillation using administrative health data sets. Using the National Ambulatory Care Reporting System,^4^ we identified patients who had visited 1 of 20 Ontario EDs with a primary diagnosis of atrial fibrillation, in the years 2009 through 2014. We excluded patients who would not benefit from^5^ or who had been taking oral anticoagulant agents within 90 days, were younger than 65 years (because we do not have comprehensive outpatient medication data for them), or had been admitted to the hospital. Data were abstracted from patient medical records for the first visit per patient. Using unique encoded identifiers, we linked the medical record data to databases held at ICES. The study was approved by the Research Ethics Board of Sunnybrook Health Sciences Centre. This report follows the Strengthening the Reporting of Observational Studies in Epidemiology (STROBE) reporting guideline.

The reference standard was medical record documentation of provision of an oral anticoagulant prescription by any physician (emergency or consultant). Thirty-two algorithms selected a priori were tested. We varied the algorithms in the following ways: (1) including oral anticoagulant prescription fills that occurred on the same day (day 0) as the patient left the ED (because some patients are sent to the ED by another clinician who may have prescribed the agent); (2) counting the number of days following discharge that patients filled a prescription; (3) excluding prescription fills that occurred after an outpatient visit with a relevant clinician (because that clinician may have written the prescription being filled); (4) excluding patients with a history of venous thromboembolism; and (5) removing patients who had seen a relevant clinician 30 days prior to the emergency visit. We calculated the sensitivity, specificity, positive predictive value (PPV), and negative predictive value (NPV), and 95% (CIs). All analyses were conducted with SAS statistical software version 9.3 (SAS Institute Inc).

## Results

Of 2015 qualifying patients, 65% had a CHADS_2_ (congestive heart failure, hypertension, age >75 years, diabetes, and stroke) score of 2 or higher. Emergency department physicians prescribed warfarin for a median of 7 days and oral anticoagulant agents for a median of 30 days. Inclusion of day 0 substantially improved sensitivity, eg, 34% of patients analyzed in algorithm 3, prescriptions filled between days 1 and 3, to 83% of patients analyzed in algorithm 2, prescriptions filled between days 0 and 3 (Table 1). Using a longer follow-up period for prescription fills lowered specificity (91% in algorithm 2 to 74% in algorithm 10, prescriptions filled between days 0 and 30, and only slightly improved sensitivity (83% to 88%).

**Table 1.** Algorithm Test Characteristics Among Patients Treated in the Emergency Department

Algorithm No.	Days in Which Oral Anticoagulant Prescription Filled	True Positive	True Negative	False Positive	False Negative	Sensitivity (95% CI)	Specificity (95% CI)	Positive Predictive Value (95% CI)	Negative Predictive Value (95% CI)	Rate, %
**Filled**										
1	0	198	1576	36	205	0.491 (0.442-0.540)	0.978 (0.971-0.985)	0.846 (0.800-0.892)	0.885 (0.870-0.900)	11.6
2	0-3	335	1466	146	68	0.831 (0.794-0.868)	0.909 (0.895-0.923)	0.696 (0.655-0.737)	0.956 (0.946-0.966)	23.9
3	1-3	137	1502	110	266	0.340 (0.294-0.386)	0.932 (0.920-0.944)	0.555 (0.493-0.617)	0.850 (0.833-0.867)	12.3
4	0-4	338	1441	171	65	0.839 (0.803-0.875)	0.894 (0.879-0.909)	0.664 (0.623-0.705)	0.957 (0.947-0.967)	25.3
5	1-4	140	1477	135	263	0.347 (0.301-0.393)	0.916 (0.902-0.930)	0.509 (0.450-0.568)	0.849 (0.832-0.866)	13.7
6	0-7	346	1374	238	57	0.859 (0.825-0.893)	0.852 (0.835-0.869)	0.592 (0.552-0.632)	0.960 (0.950-0.970)	29.0
7	1-7	148	1410	202	255	0.367 (0.320-0.414)	0.875 (0.859-0.891)	0.423 (0.371-0.475)	0.847 (0.830-0.864)	17.4
8	0-14	350	1297	315	53	0.868 (0.835-0.901)	0.805 (0.786-0.824)	0.526 (0.488-0.564)	0.961 (0.951-0.971)	33.0
9	1-14	152	1333	279	251	0.377 (0.330-0.424)	0.827 (0.809-0.845)	0.353 (0.308-0.398)	0.842 (0.824-0.860)	21.4
10	0-30	356	1189	423	47	0.883 (0.852-0.914)	0.738 (0.717-0.759)	0.457 (0.422-0.492)	0.962 (0.951-0.973)	38.7
11	1-30	158	1225	387	245	0.392 (0.344-0.440)	0.760 (0.739-0.781)	0.290 (0.252-0.328)	0.833 (0.814-0.852)	27.1
**Filled Prior to Any Appointment With a Family Physician, Cardiologist, or Internist**										
12	0-14	264	1560	52	139	0.655 (0.609-0.701)	0.968 (0.959-0.977)	0.835 (0.794-0.876)	0.918 (0.905-0.931)	15.7
13	0-30	272	1557	55	131	0.675 (0.629-0.721)	0.966 (0.957-0.975)	0.832 (0.791-0.873)	0.922 (0.909-0.935)	16.2
14	0-30^a^	272	1557	55	131	0.675 (0.629-0.721)	0.966 (0.957-0.975)	0.832 (0.791-0.873)	0.922 (0.909-0.935)	16.2
**Filled Prior to Any Appointment With a Family Physician, Cardiologist, or Internist and Had No Appointments 30 d Prior to ED Visit**										
15	0-30	98	1597	15	305	0.243 (0.201-0.285)	0.991 (0.986-0.996)	0.867 (0.804-0.930)	0.840 (0.824-0.856)	5.6
**Filled Prior to Any Appointment With Their Family Physician, Cardiologist, or Internist**										
16	0-30	236	1567	45	167	0.586 (0.538-0.634)	0.972 (0.964-0.980)	0.840 (0.797-0.883)	0.904 (0.890-0.918)	14.0

Abbreviation: ED, emergency department.

^a^ Includes a nephrologist.

After restricting to patients who filled a prescription before they saw another clinician, the number of false-positive results (ie, prescriptions filled that were not written in the ED) decreased. Thus, specificity improved from 74% in algorithm 10 to 97% in algorithm 13; however, sensitivity fell from 88% to 68%. Removal of patients with an outpatient visit prior to the ED visit (eg, algorithm 15) further increased specificity (99%) but decreased sensitivity (24%). Excluding patients with a prior venous thromboembolism resulted in minimal change (Table 2).

**Table 2.** Algorithm Test Characteristics Among Patients Treated in the Emergency Department With No Venous Thromboembolism Diagnosis in the Previous 5 Years

Algorithm No.	Days in Which Oral Anticoagulant Prescription Filled	True- Positive	True Negative	False Positive	False Negative	Sensitivity (95% CI)	Specificity (95% CI)	Positive Predictive Value (95% CI)	Negative Predictive Value (95% CI)	Rate, %
**Filled**										
17	0	194	1524	35	197	0.496 (0.446-0.546)	0.978 (0.971-0.985)	0.847 (0.800-0.894)	0.886 (0.871-0.901)	11.7
18	0-3	325	1418	141	66	0.831 (0.794-0.868)	0.910 (0.896-0.924)	0.697 (0.655-0.739)	0.956 (0.946-0.966)	23.9
19	1-3	131	1453	106	260	0.335 (0.288-0.382)	0.932 (0.920-0.944)	0.553 (0.490-0.616)	0.848 (0.831-0.865)	12.2
20	0-4	328	1394	165	63	0.839 (0.803-0.875)	0.894 (0.879-0.909)	0.665 (0.623-0.707)	0.957 (0.947-0.967)	25.3
21	1-4	134	1429	130	257	0.343 (0.296-0.390)	0.917 (0.903-0.931)	0.508 (0.448-0.568)	0.848 (0.831-0.865)	13.5
22	0-7	336	1329	230	55	0.859 (0.825-0.893)	0.852 (0.834-0.870)	0.594 (0.554-0.634)	0.960 (0.950-0.970)	29.0
23	1-7	142	1364	195	249	0.363 (0.315-0.411)	0.875 (0.859-0.891)	0.421 (0.368-0.474)	0.846 (0.828-0.864)	17.3
24	0-14	340	1255	304	51	0.870 (0.837-0.903)	0.805 (0.785-0.825)	0.528 (0.489-0.567)	0.961 (0.951-0.971)	33.0
25	1-14	146	1290	269	245	0.373 (0.325-0.421)	0.827 (0.808-0.846)	0.352 (0.306-0.398)	0.840 (0.822-0.858)	21.3
26	0-30	346	1150	409	45	0.885 (0.853-0.917)	0.738 (0.716-0.760)	0.458 (0.422-0.494)	0.962 (0.951-0.973)	38.7
27	1-30	152	1185	374	239	0.389 (0.341-0.437)	0.760 (0.739-0.781)	0.289 (0.250-0.328)	0.832 (0.813-0.851)	27.0
**Filled Prior to Any Appointment With a Family Physician, Cardiologist, or Internist**										
28	0-14	257	1508	51	134	0.657 (0.610-0.704)	0.967 (0.958-0.976)	0.834 (0.792-0.876)	0.918 (0.905-0.931)	15.8
29	0-30	265	1505	54	126	0.678 (0.632-0.724)	0.965 (0.956-0.974)	0.831 (0.790-0.872)	0.923 (0.910-0.936)	16.4
30	0-30^a^	265	1505	54	126	0.678 (0.632-0.724)	0.965 (0.956-0.974)	0.831 (0.790-0.872)	0.923 (0.910-0.936)	16.4
**Filled Prior to Any Appointment With a Family Physician, Cardiologist, or Internist and Had No Appointments 30 d Prior to ED Visit**										
31	0-30	95	1544	15	296	0.243 (0.200-0.286)	0.990 (0.985-0.995)	0.864 (0.800-0.928)	0.839 (0.822-0.856)	5.6
**Filled Prior to Any Appointment With Their Family Physician, Cardiologist, or Internist**										
32	0-30	229	1515	44	162	0.586 (0.537-0.635)	0.972 (0.964-0.980)	0.839 (0.795-0.883)	0.903 (0.889-0.917)	14.0

Abbreviation: ED, emergency department.

^a^ Includes a nephrologist.

Algorithm 2 maximized specificity (91%; 95% CI, 90%-92%) while maintaining reasonable sensitivity (83%; 95% CI, 79%-87%) (PPV, 70%; 95% CI, 66%-74%, NPV, 96%; 95% CI, 95%-97%). Algorithm 13, prescription filled prior to seeing another relevant clinician, provided high specificity (97%; 95% CI, 96%-98%).

Limitations include using medical record documentation of ED prescription provision as reference standard, and our results may not apply to patients younger than 65 years.

We derived several algorithms that identify ED oral anticoagulant prescriptions in a large health data set. Depending on project goals, an algorithm can be selected to optimize specificity, sensitivity, PPV, or NPV.
